# Group B Streptococcal Septic Arthritis of the Shoulder and Potential Association with Pelvic Examination and PAP Smear

**DOI:** 10.1155/2016/5294517

**Published:** 2016-02-14

**Authors:** William E. Daner, Brett D. Meeks, William C. Foster, Norman D. Boardman

**Affiliations:** Department of Orthopaedic Surgery, Virginia Commonwealth University Health System, Richmond, VA 23298, USA

## Abstract

Group B streptococcal (GBS) infection of a native joint in a nonpregnant adult is uncommon. While many women are colonized with this flora, it rarely becomes pathogenic in its adult host. GBS associated joint infections have been reported, most of which have been related to hematogenous seeding from unknown sources. To our knowledge, there are no published case reports of a GBS joint infection in association with a pelvic exam and Papanicolaou (PAP) smear. In this case report, we present a case of GBS sepsis of a native shoulder, possibly resulting from a routine pelvic exam and PAP smear.

## 1. Introduction

Glenohumeral joint sepsis accounts for 10–15% of all infected joints, ranking third behind knee and hip infections [1, 2]. The most common route of joint sepsis is via hematogenous seeding, less commonly as a result of introduction of bacteria at either the time of a prior surgery or joint injection [3–5].* Staphylococcus aureus* is the most common pathogen in septic shoulders, followed by* Streptococcus pyogenes* and Gram negative organisms [3, 6–9].* Propionibacterium acnes* and* Staphylococcus epidermidis* are more common pathogens in shoulders following surgery and are rarely found in virgin shoulders. Group B streptococcus (GBS) is a rare cause of native joint sepsis.

Although GBS is most commonly considered a neonatal pathogen, the incidence of invasive GBS infections in nonpregnant adults has significantly increased since the 1990s, and joint infections comprise 5.4% of all GBS infections [10]. We present a case of GBS sepsis of a native shoulder, possibly resulting from a routine pelvic exam and Papanicolaou (PAP) smear.

## 2. Case Report

**Figure 1:**
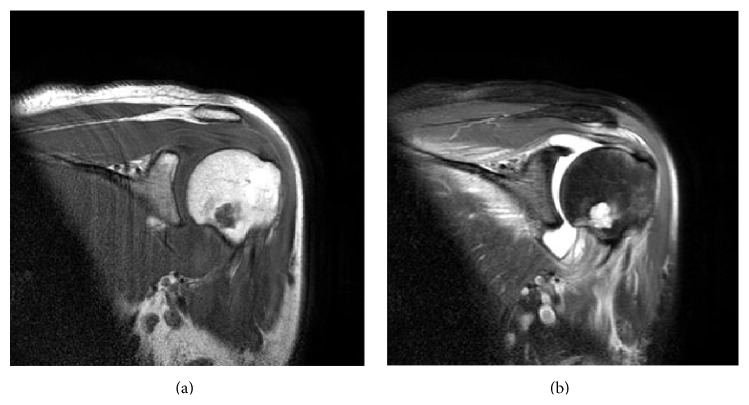
Selected MRI T1 (a) and T2 (b) sagittal cuts demonstrating the large joint effusion and proximal humerus lesion.

The patient is a 30-year-old sexually active female referred from an outside provider with two weeks of left shoulder pain. She had initially attributed the pain to an episode of “rough housing” with her boyfriend. However, the pain progressed to the point that she was unable to tolerate even minimal movement of the joint. She reported intermittent fevers and an episode of vaginal discharge over several weeks, as well as a routine pelvic exam and PAP smear 8 weeks prior to presentation. She did not have any skin trauma and had no significant medical history, diabetes, immunocompromise, or recent dental visits. An outside magnetic resonance imaging (MRI) study demonstrated a 1.2 cm benign appearing cartilaginous lesion in her proximal humerus with a large joint effusion (Figure 1). Erythrocyte sedimentation rate was 108 mm/hr and C-reactive protein was 15.2 mg/dL. Shoulder aspiration yielded a synovial fluid white blood cell count of >48,000, with 97% neutrophils.

**Figure 2:**
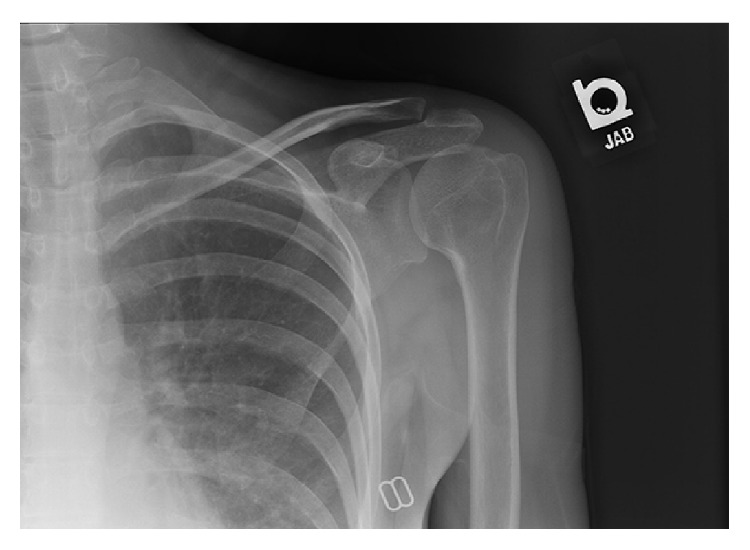
Anteroposterior radiograph at 6 months post-op.

She underwent open irrigation and debridement with biopsy of the proximal humerus lesion. Purulent bloody fluid was encountered in the joint, though the articular cartilage appeared to be in good condition. Biopsy of the lesion was consistent with the clinical impression of enchondroma. Intraoperative and preoperative cultures grew GBS; no bacteria were isolated from blood or urine cultures. Due to a penicillin allergy, she was treated with 4 weeks of IV ceftriaxone (due to penicillin allergy), followed by two weeks of oral cefpodoxime. She developed stiffness in her shoulder and required a regimen of passive and active motion exercises with physical therapy. By her 6-month follow-up visit, she was pain-free and had recovered full range of motion in her shoulder. A follow-up radiograph is shown in Figure 2.

## 3. Discussion

GBS infection of a native joint in a nonpregnant adult is uncommon. It is more commonly considered a neonatal pathogen transmitted during child bearing from a colonized mother. In addition to the vagina and cervix, GBS may also be found to colonize the skin, genitourinary tract, pharynx, and rectum [11–13]. GBS colonization rates in healthy, young adults have been reported to be as high as 34% in women and 20% in men [12, 14]. Loss of “barrier protection” from a chronically colonized site could lead to hematogenous spread [15]. Previously suggested routes of entry have included genitourinary infections, tonsillitis, skin infection, GI following resection of a colon tumor, and the oropharynx during dental procedures [16, 17]. Kumar et al. reported a case of maternal GBS septic hip following a vaginal childbirth in a 30-year-old woman [18]. GBS infections have also been found to be associated with immunocompromised states, diabetes, liver disease in males, and cervical carcinoma in females [10, 16, 19].

The incidence of GBS infections has doubled since the early 1990s [10], possibly the result of the increasing prevalence of more virulent strains of GBS [15]. The most common presentation of nonpregnancy related GBS infection is bacteremia without a known source. Skin and soft tissue infections are the second most common presentation, followed by pneumonia and less common infections, such as endocarditis and meningitis [10]. Joint sepsis comprises 5.4% of invasive GBS infections [10]. Of native joints infected with GBS, the knee is the most commonly reported in the literature (36% of cases), followed by shoulder (25%), hip (16%) [16], the small joints of the hands or feet [19], and less commonly spondylodiscitis [15] and sacroilitis [20]. GBS has also been isolated from hip and knee prosthetic joint infections (PJI), with 75% of GBS PJI occurring greater than 3 months after surgery. This time course may indicate that the route of infection is often via hematogenous seeding [21].

Most patients afflicted with GBS infection of native joints are of middle age or older; only 11% of patients in published case reports were less than 40 years of age [16]. In addition to pain in the affected joint, the acutely infected patient may present febrile with GBS bacteremia that may be isolated in blood culture. Nonspecific inflammatory markers, such as CRP and ESR, are also typically elevated. Over half of reported infections are monoarticular (68%), but providers should be cognizant of the high risk of additional sites of infection. Nolla et al. reported the frequency of a concomitant GBS infection elsewhere to be 31%. Other potential sites of infection include vertebral osteomyelitis (11%), urinary tract infections (7%), endocarditis (4%), and endophthalmitis (4%) [16].

Group B streptococcus is an uncommon cause of septic arthritis but is increasing in frequency. The source of infection may be unclear. The infectious disease service hypothesized that our patient's source was a routine gynecologic pelvic exam and PAP smear 8 weeks prior to presentation. Hematogenous spread from the GU tract to the shoulder in our patient cannot be proven, but no other source was identified. Certainly, the benefit of a screening PAP smear far outweighs any potential risk of bacteremia and joint sepsis. With urgent irrigation and debridement and a prolonged course of antibiotics, our patient made a full recovery.

## 4. Conclusion

GBS is a rare cause of joint sepsis, but one whose prevalence may be increasing. Screening gynecologic pelvic exams and PAP smears could theoretically produce bacteremia and should be considered as a potential source of joint sepsis in otherwise healthy, young, nonimmunocompromised women. However, benefits of such gynecologic screening exams far outweigh any potential risk of bacteremia and joint sepsis. Early identification and appropriate, timely management of GBS shoulder infections are paramount to a good outcome.
